# Supplementary material for: The megabase-scale crossover landscape is largely independent of sequence divergence
Source: Nat Commun. 2022 Jul 2;13:3828. doi: 10.1038/s41467-022-31509-8 (PMC9250513; doi:10.1038/s41467-022-31509-8)
Supplement: Supplementary file 1 — Supplementary Information [file 41467_2022_31509_MOESM1_ESM.pdf]

**Supplementary Information**

# The megabase-scale crossover landscape is largely independent of sequence divergence

Qichao Lian, Victor Solier, Birgit Walkemeier, Stéphanie Durand, Bruno Huettel, Korbinian Schneeberger*, Raphael Mercier*

*Corresponding author: Raphael Mercier (mercier@mpipz.mpg.de), Korbinian Schneeberger (schneeberger@mpipz.mpg.de)

**Supplementary Table 1. Summary of EMS-induced mutations in Arabidopsis inbred lines**

| Arabidopsis accession | F1* | Sequencing depths | Num. of variations after filtering | Num. of phased mutations | % of phased mutations | Num. of phased mutations per F1 |
|---|---|---|---|---|---|---|
| Col | 4314_A | 65.72 | 838 | 838 | 100 | 391 |
| Col | 4314_B | 44 | 955 | 955 | 100 | 448 |
| Col | 4314_E | 64.17 | 982 | 982 | 100 | 460 |
| Col | 4314_F | 62.27 | 953 | 953 | 100 | 449 |
| L*er* | 4560_B | 16.88 | 512 | 509 | 99.41 | 239 |
| L*er* | 4560_C | 16.22 | 471 | 471 | 100 | 214 |
| L*er* | 4560_D | 13.05 | 539 | 536 | 99.44 | 252 |
| L*er* | 4560_E | 17.03 | 526 | 521 | 99.05 | 243 |

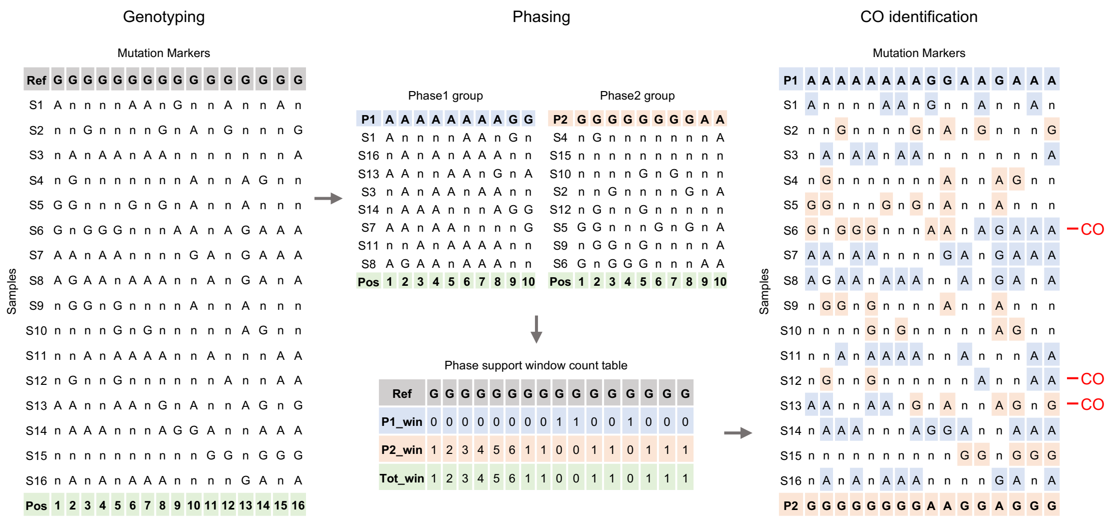


**Supplementary Figure 1. Mutation marker phasing and CO identification.**
The EMS-induced mutation markers were first genotyped in each F1 population. Then, in each sliding window of markers, F1 samples were clustered into two groups, corresponding to the two different phases (P1 and P2, alleles coloured blue and orange). After phasing, the count table was generated, indicating the number of supported windows of each phase on each marker position. With this, uncovered and mis-genotyped markers were imputed and corrected. Finally, COs can be identified as consistent switches of phase. "G" and "A" represent markers that genotyped as reference and mutant alleles, "n" represents markers not covered by sequencing reads.

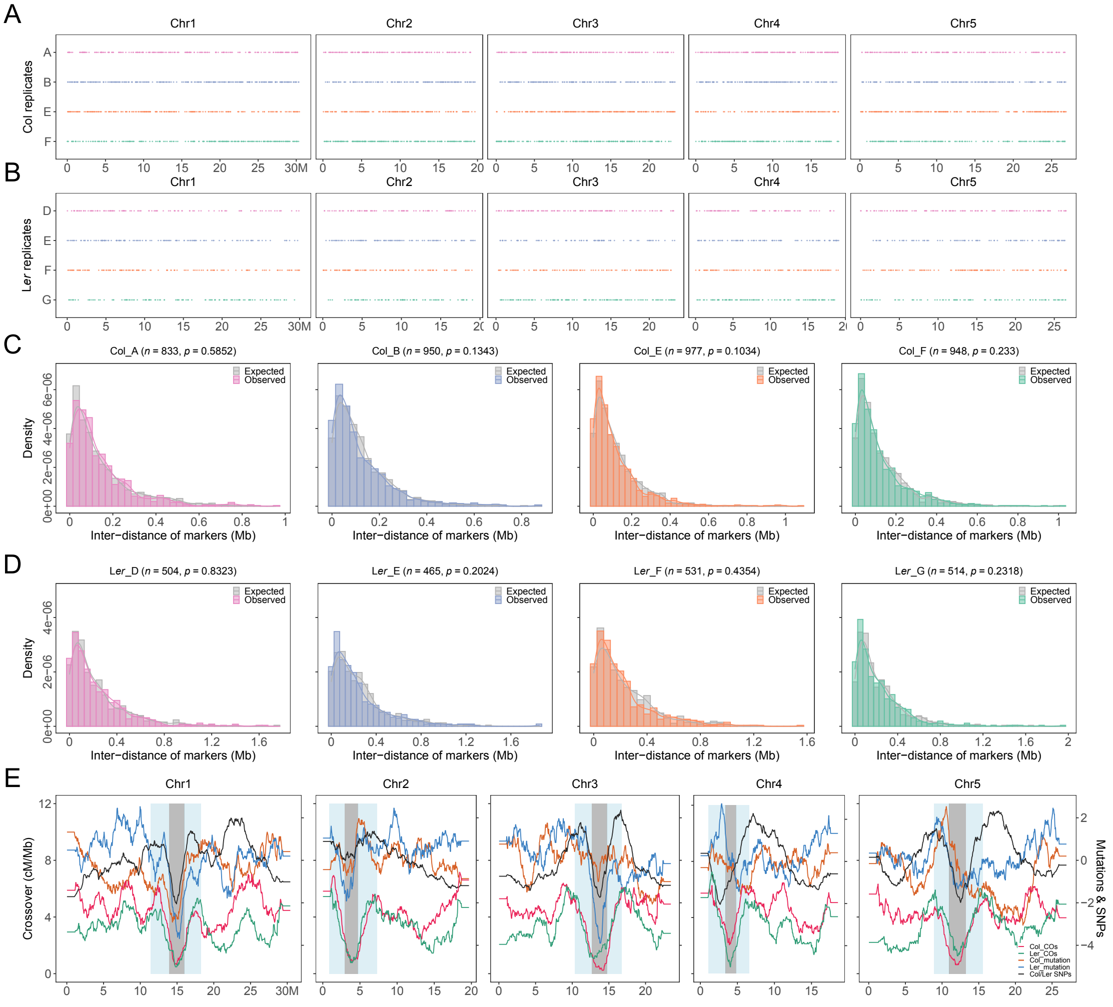


**Supplementary Figure 2. The chromosomal distribution of phased EMS-induced mutation markers in Col and L*er*.**

(A-B) The distribution of phased EMS-induced mutation markers along chromosomes in each replicate of Col and L*er* populations, respectively. (C-D) The distribution of distance between two nearby mutation markers in each replicate population of Col and L*er*. The grey bar shows the inter-distance distribution of mutation markers, supposing they are randomly distributed along chromosomes. The number of mutation markers and the p-value of two-sided Mann-Whitney test between the expected and observed distributions are shown in parentheses. (E) Sliding window-based distribution (window size 2 Mb, step size 50 kb) of COs and mutation markers in Col and L*er*, and SNPs between Col and L*er*. Source Data are provided as a Source Data file.

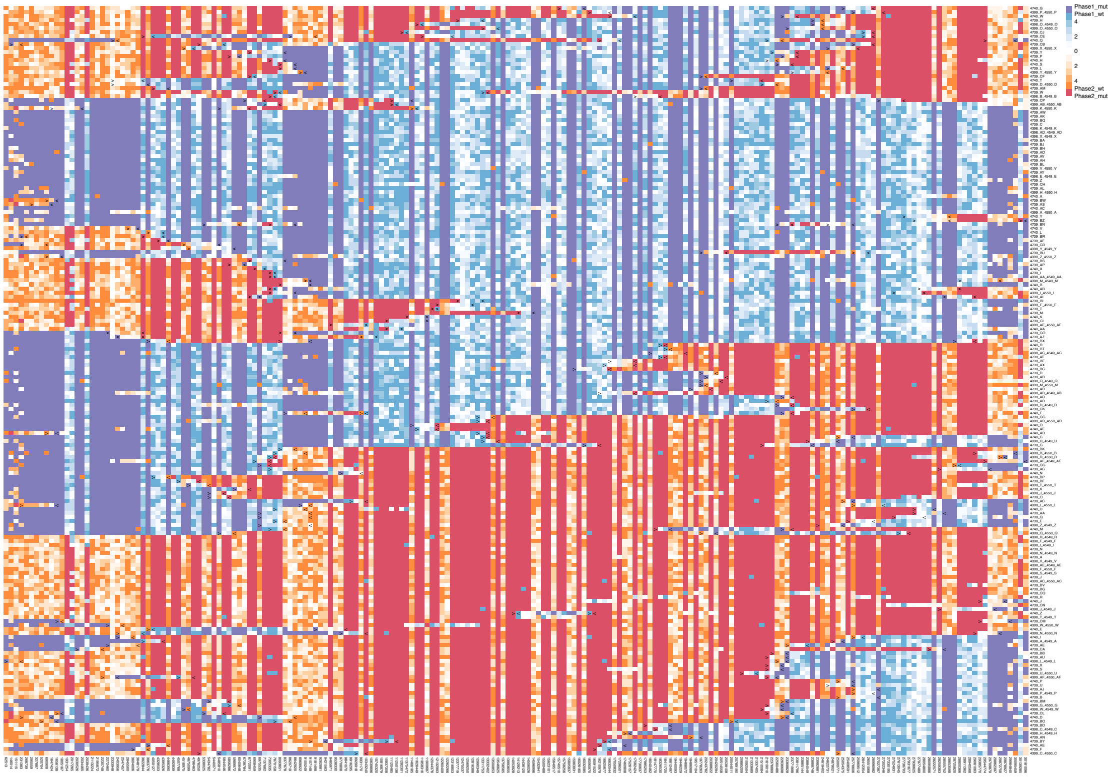


**Supplementary Figure 3. The phased genotype map of mutation markers on chromosome 1 in Col A population.**
Each line is a F1 individual, each column a marker. As the F1 results from reciprocal crosses between independent F1*s, a marker in a given F1* can be either heterozygous or wild-type. Dark purple and dark red indicate the detection of mutant reads (phase 1 and phase 2, respectively), unambiguously scored as a heterozygote. When only wild-type reads are detected, the number of reads is indicated with a colour scale. (phase 1 and phase 2 are coloured blue and orange, respectively. Maximum was set as 5 manually). The identified COs are marked with pairs of black arrows. Source Data are provided as a Source Data file.

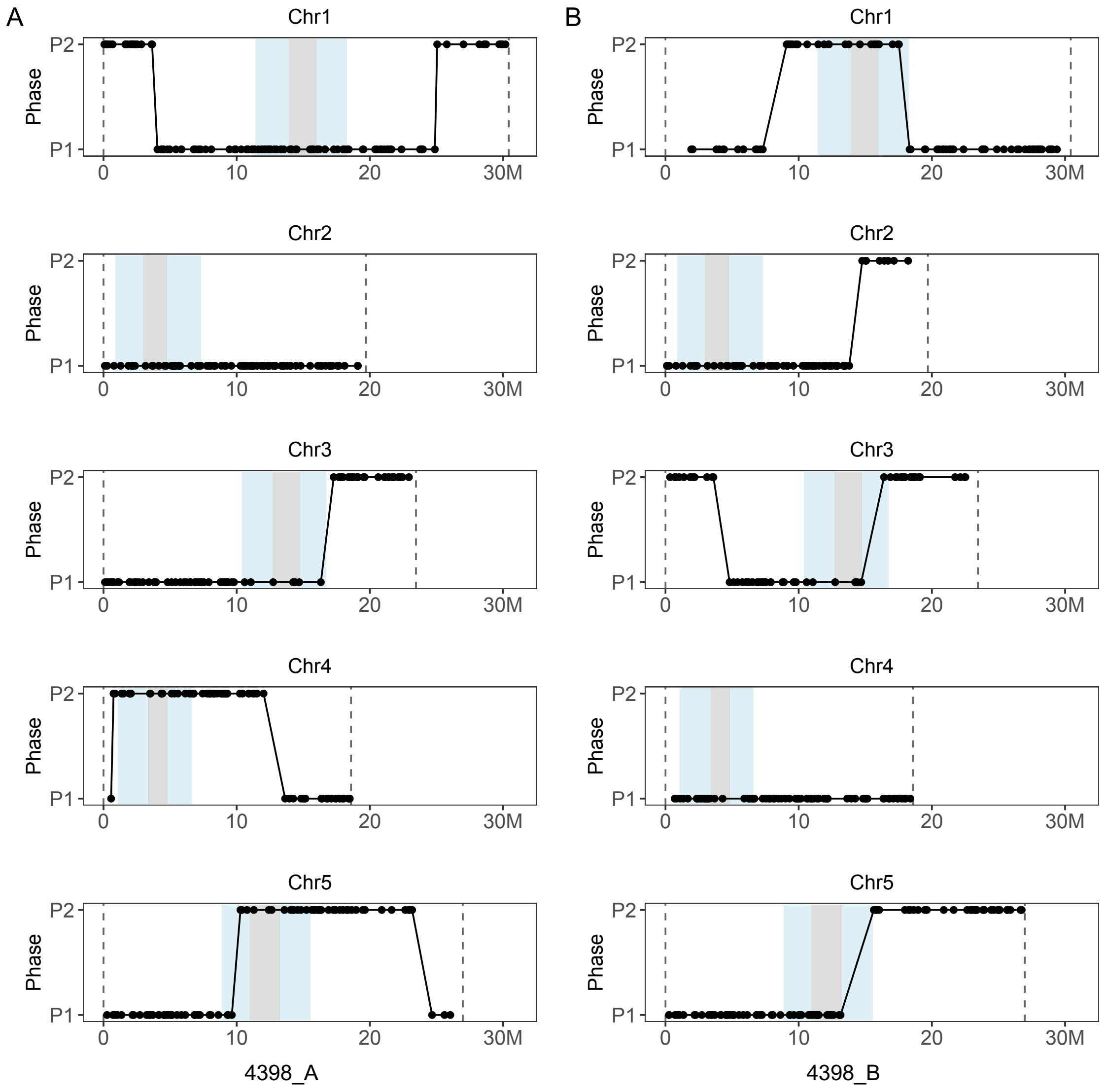


**Supplementary Figure 4. The genome-wide phase map of mutation markers in two samples of Col A population.**

Graphical representation of phases shows CO identification in 4398_A (A) and 4398_B (B). The bounds of chromosomes are indicated by vertical dashed lines. The pericentromeric and centromeric regions are indicated by grey and blue shading, separately. Source Data are provided as a Source Data file.

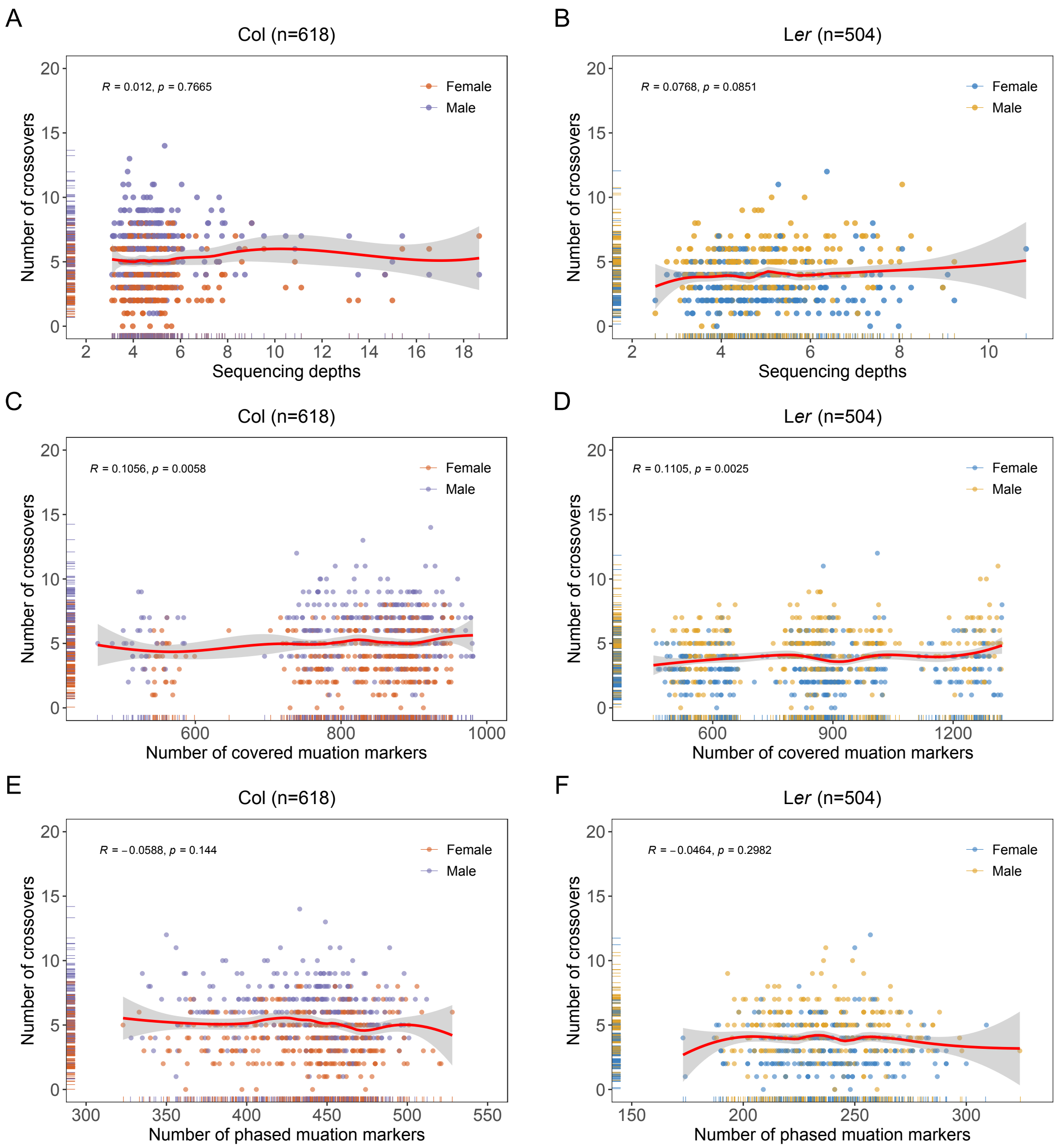


**Supplementary Figure 5. Correlation analysis of CO numbers, sequencing depths and number of covered and phased mutation markers in each replicate population of Col and L*er*.**

(A-B) The correlation analysis between CO numbers and sequencing depths in Col and L*er*, respectively. (C-D) The correlation analysis between CO numbers and number of covered mutation markers in Col and L*er*, respectively. (E-F) The correlation analysis between CO numbers and number of phased mutation markers in Col and L*er*, respectively. Pearson's correlation coefficient and p-value are shown for each test. The line was fitted by loess method, with 95% confidence interval. Source Data are provided as a Source Data file.

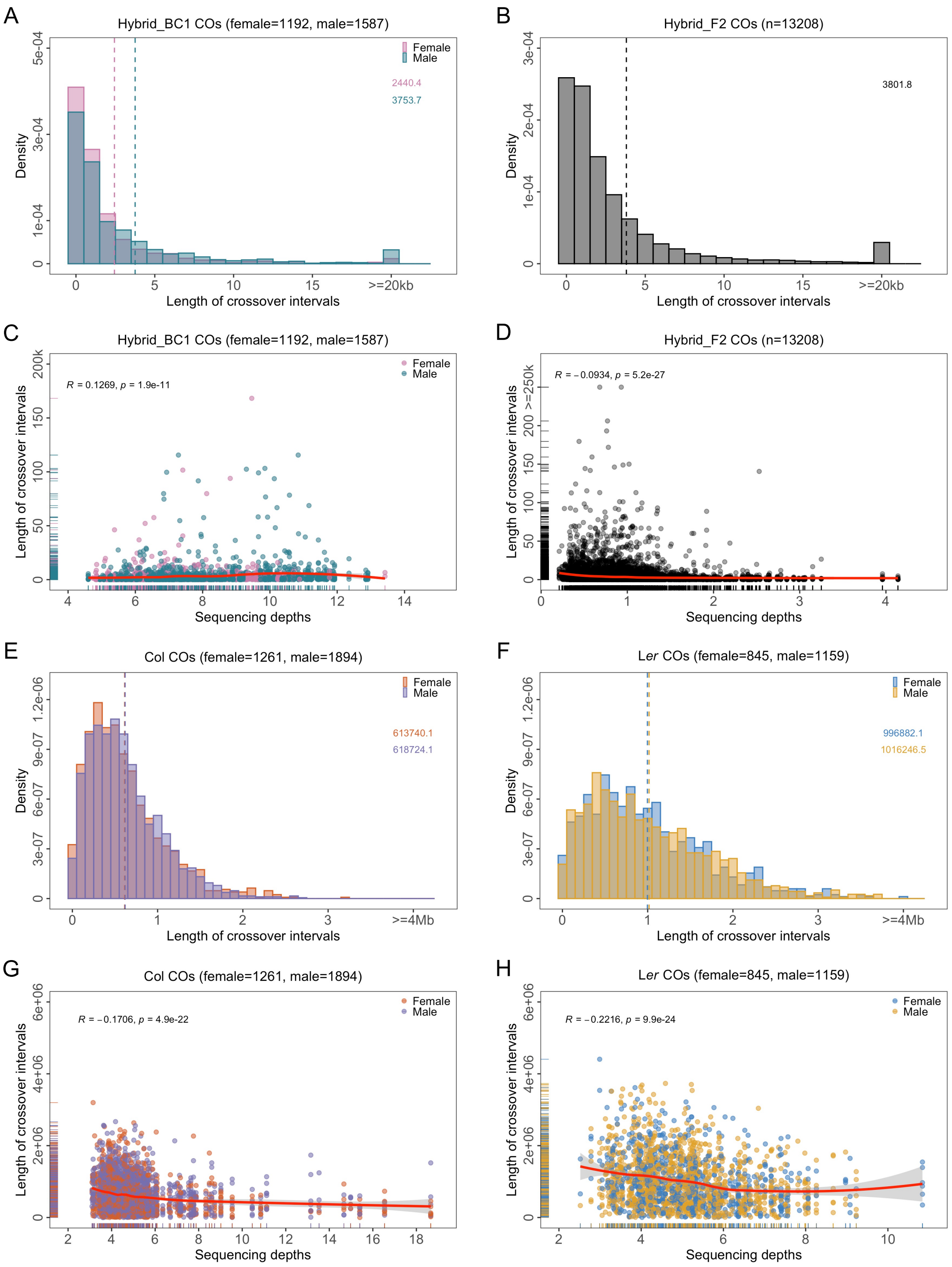


**Supplementary Figure 6. The distribution of interval length of COs and correlation analysis with sequencing depths in Col, L*er* and hybrids.**

The distribution of CO interval length (A-B, E-F), correlation analysis between CO interval length and sequencing depths (C-D, G-H) in F1, F2 hybrids, Col and L*er*, respectively. Pearson's correlation coefficient and p-value are shown for each test. The line was fitted by loess method, with 95% confidence interval. Source Data are provided as a Source Data file.

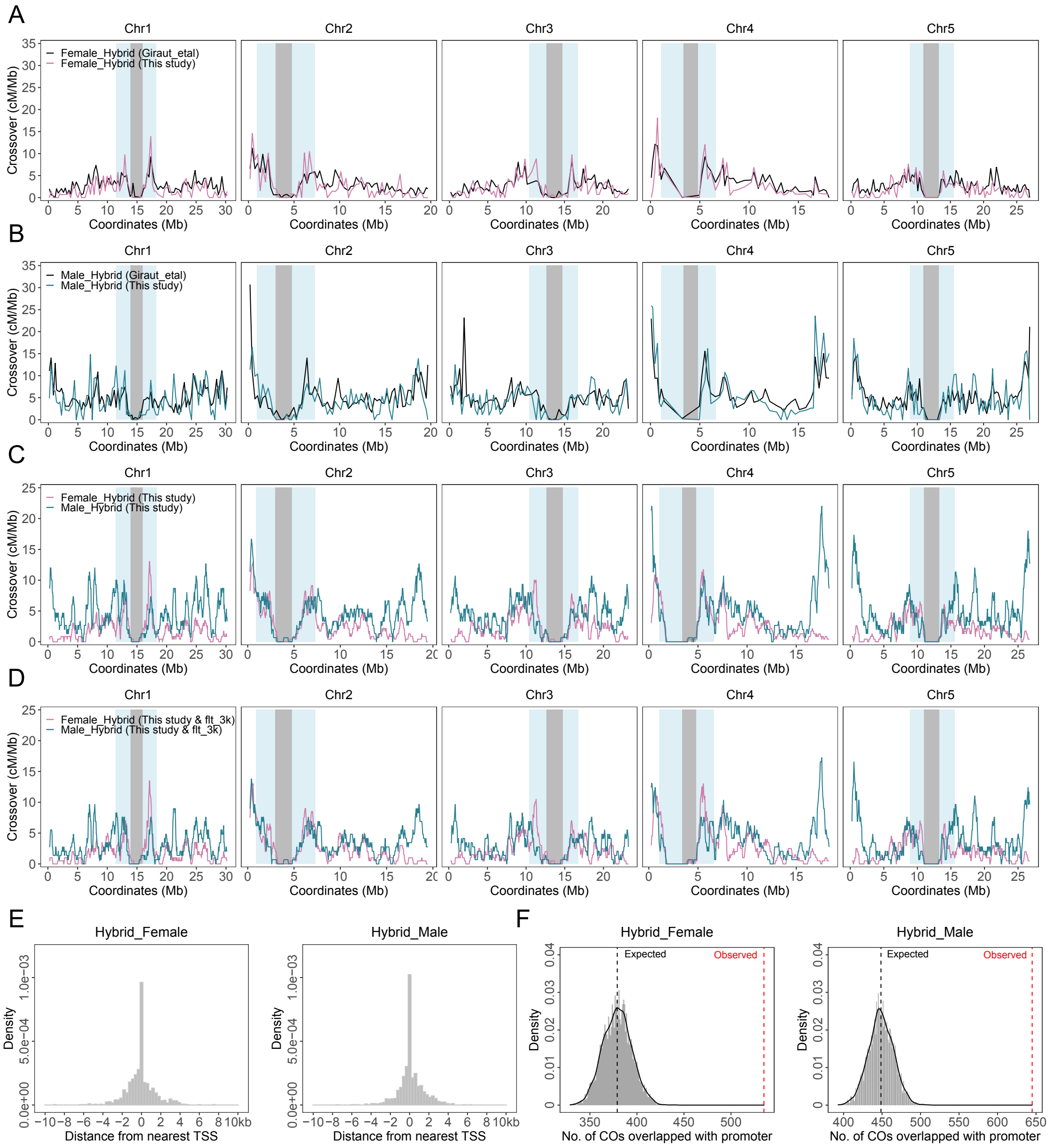


**Supplementary Figure 7. The chromosomal distribution of COs in Col/L*er* F1 hybrids and relationship to genome features.**

(A-B) Distribution of COs in female (pink) and male (blue) meiosis of F1 hybrids in this study compared with Giraut et al. (black). The exact same windows were used to allow comparison with Giraut et al. (C) Sliding window-based distribution (window size 500 kb, step size 50 kb) of COs in female and male meiosis of F1 hybrids (this study). (D) Sliding window-based distribution (window size 500 kb, step size 50 kb) of high-resolution COs (COs were filtered for precision of localization <= 3 kb) in female and male meiosis of F1 hybrids (this study). (E) Distribution of distance of high-resolution COs from nearest TSS. (F) Permutation tests (5,000 shuffling) were performed to evaluate the overlap between promoter regions of proteins encoding genes and high-resolution COs in F1 hybrids. Source Data are provided as a Source Data file.

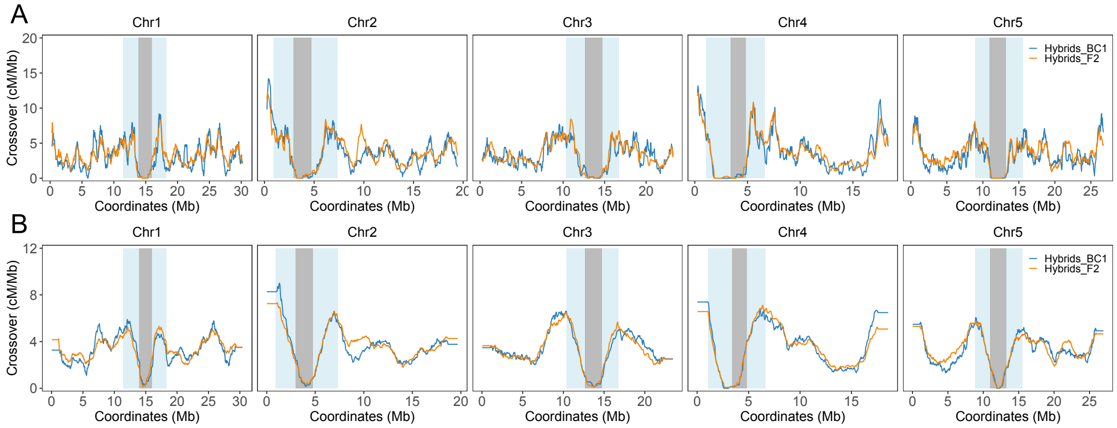


**Supplementary Figure 8. The distribution of CO frequency across genomes in Col/L*er* detected in backcrosses (F1, this study) and in selfing (F2, Rowan et al) populations.** Comparison was done using a window size of 500 kb and step size of 50 kb (A) or a window size of 2 Mb and step size of 50 kb (B). Female and male data (this study, blue) were merged to be comparable with the selfing data (Rowan et al., orange). Source Data are provided as a Source Data file.

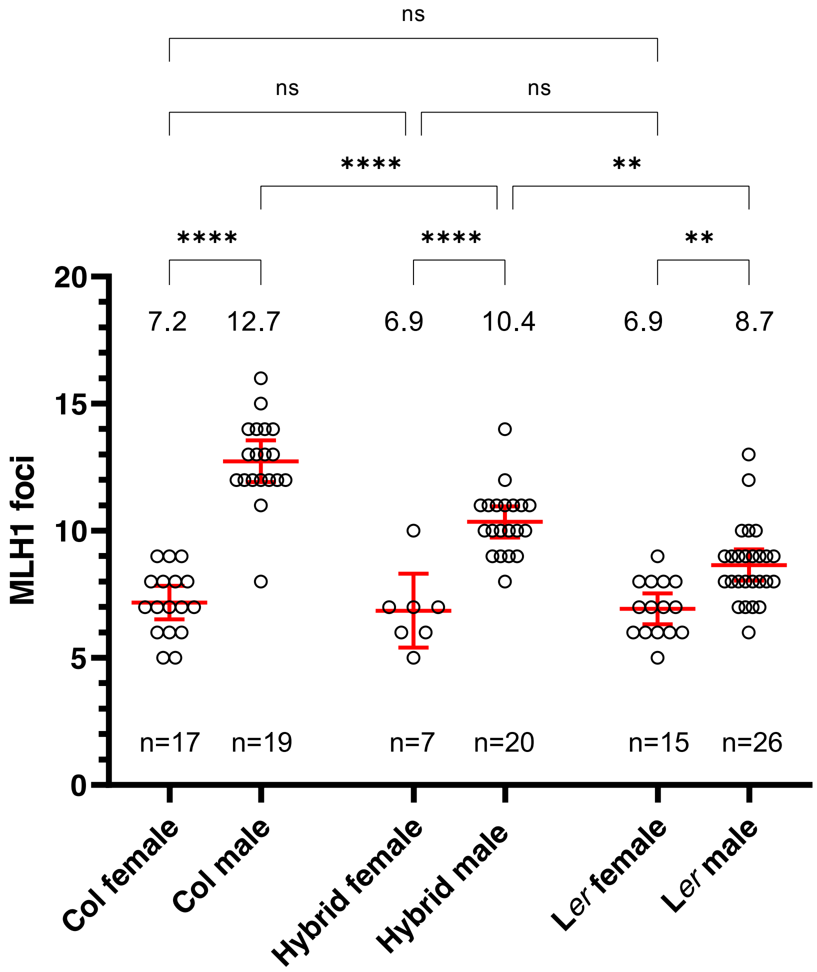


**Supplementary Figure 9. MLH1 foci counts in female and male meiocytes of Col, L*er*, and Col/L*er* hybrids.**

The number of MLH1/HEI10 per cell is shown for each genotype and each sex. Bars represent mean +/- 95% intervals of confidence. The average number of foci is indicated, and differences were tested using two-sided ANOVA with Sidak correction for multiple testing. For the P values, Col female vs. Col male <0.0001, L*er* female vs. L*er* male = 0.0027, Hybrid female vs. Hybrid male <0.0001, Col male vs. Hybrid male <0.0001, L*er* male vs. Hybrid male = 0.001, Col female vs. Hybrid female = 0.9996, L*er* female vs. Hybrid female >0.9999, Col female vs. L*er* female = 0.9997. Source Data are provided as a Source Data file.

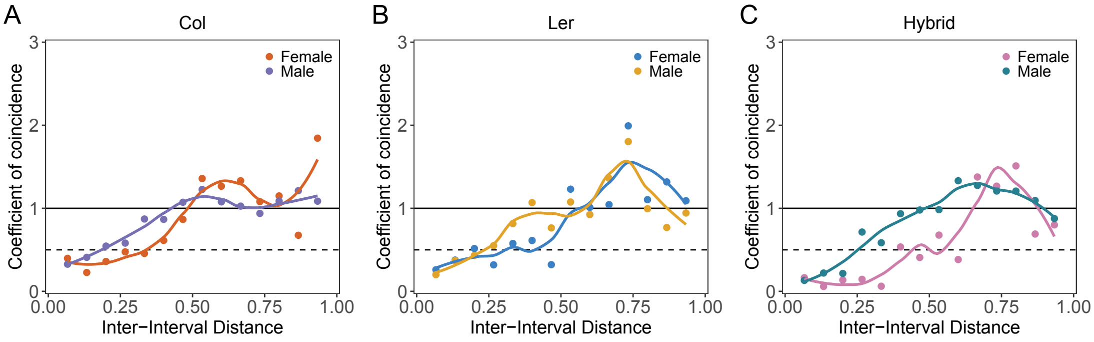


**Supplementary Figure 10. Comparison of the CoC curves between female and male meiosis in Col, L*er* and F1 hybrids.**

The CoC Curves in female and male populations of Col (A), L*er* (B) and F1 hybrids (C), respectively. Chromosomes were divided into 15 intervals, and the mean coefficients of coincidence (CoC = observed number of coincident occurrence of COs in both intervals/expected number according to CO frequency in each interval) were calculated for pairs of intervals separated by a certain distance (proportion of chromosome length). A CoC lower than 1 indicates the presence of crossover interference. Source Data are provided as a Source Data file.

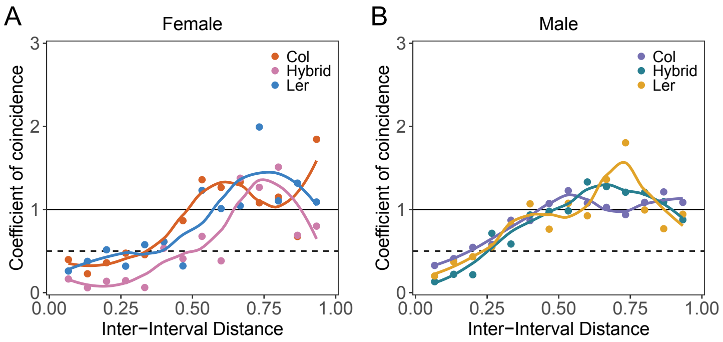


**Supplementary Figure 11. Comparison of the CoC curves between Col, L*er* and F1 hybrids in female and male meiosis.**

The CoC Curves in female (A) and male (B) populations of Col, L*er* and F1 hybrids, respectively. Chromosomes were divided into 15 intervals, and the mean coefficients of coincidence (CoC = observed number of coincident occurrence of COs in both intervals/expected number according to CO frequency in each interval) were calculated for pairs of intervals separated by a certain distance (proportion of chromosome length). A CoC lower than 1 indicates the presence of crossover interference. Source Data are provided as a Source Data file.

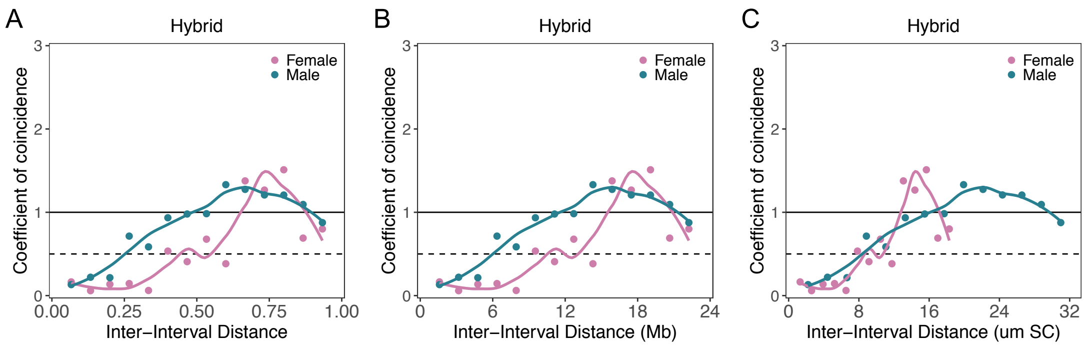


**Supplementary Figure 12. Comparison of the CoC curves between female and male meiosis F1 hybrids.**

The CoC Curves in female and male populations of F1 hybrids, on scale of chromosome length of proportional (A), Mb (B) and um SC (C), respectively. Chromosomes were divided into 15 intervals, and the mean coefficients of coincidence (CoC = observed number of coincident occurrence of COs in both intervals/expected number according to CO frequency in each interval) were calculated for pairs of intervals separated by a certain distance (in scale of proportion of chromosome length, Mb and μm SC). A CoC lower than 1 indicates the presence of crossover interference. Source Data are provided as a Source Data file.

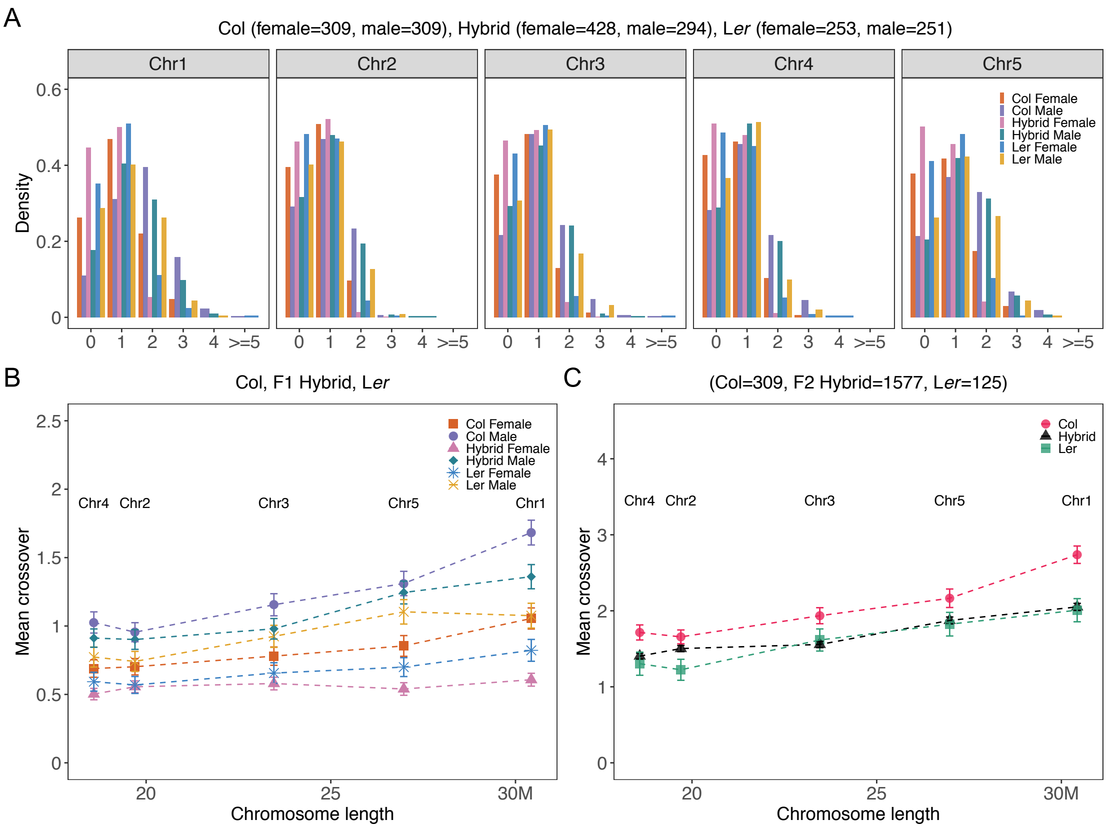


**Supplementary Figure 13. CO number distribution and correlation analysis with chromosome lengths in female and male of Col, L*er* and hybrids.**

(A) Distribution of CO numbers per chromosome and per gamete in female and male meiosis of Col, L*er* and F1 hybrids. (B) Mean numbers of COs per chromosome per gamete versus chromosome size in Mb, in female and male Col, L*er* and F1 hybrids. Pearson's correlation analysis of relationship between CO number and chromosome length: Col female r = 0.96, P = 0.008, Col male = 0.96, P = 0.011, L*er* female r = 0.97, P = 0.007, L*er* male r = 0.94, P = 0.017, Hybrid female r = 0.71, P = 0.18, Hybrid male r = 0.97, P = 0.007. (C) Pearson's correlation analysis of relationship between CO number and chromosome length in Col, L*er* pseudo F2, and hybrids F2 (Rowan et al) (Col r = 0.96, P = 0.01, L*er* r = 0.98, P = 0.003, Hybrid r = 0.98, P = 0.004). Source Data are provided as a Source Data file.

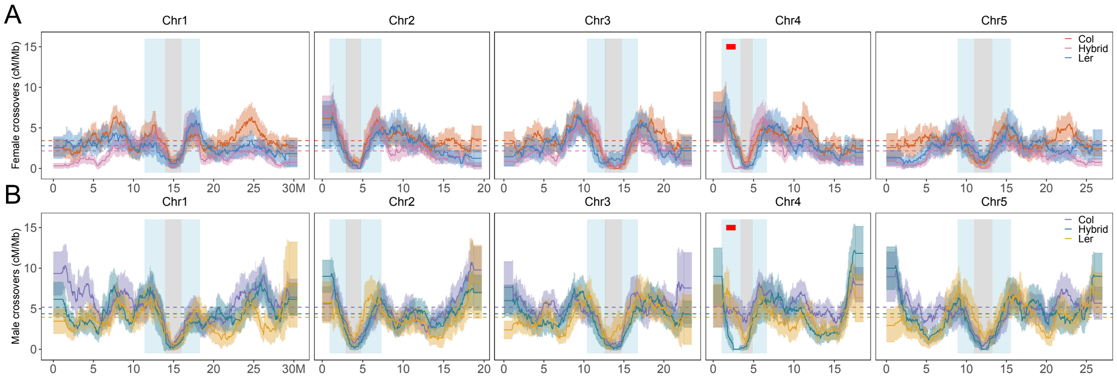


**Supplementary Figure 14. Comparison of the chromosomal distribution of COs between Col, L*er* and F1 hybrids in female and male meiosis.**

The sliding window-based distribution (window size 2 mb, step size 50 kb) of COs in female (A) and male (B) meiosis of Col, L*er* and hybrids individually. Source Data are provided as a Source Data file.

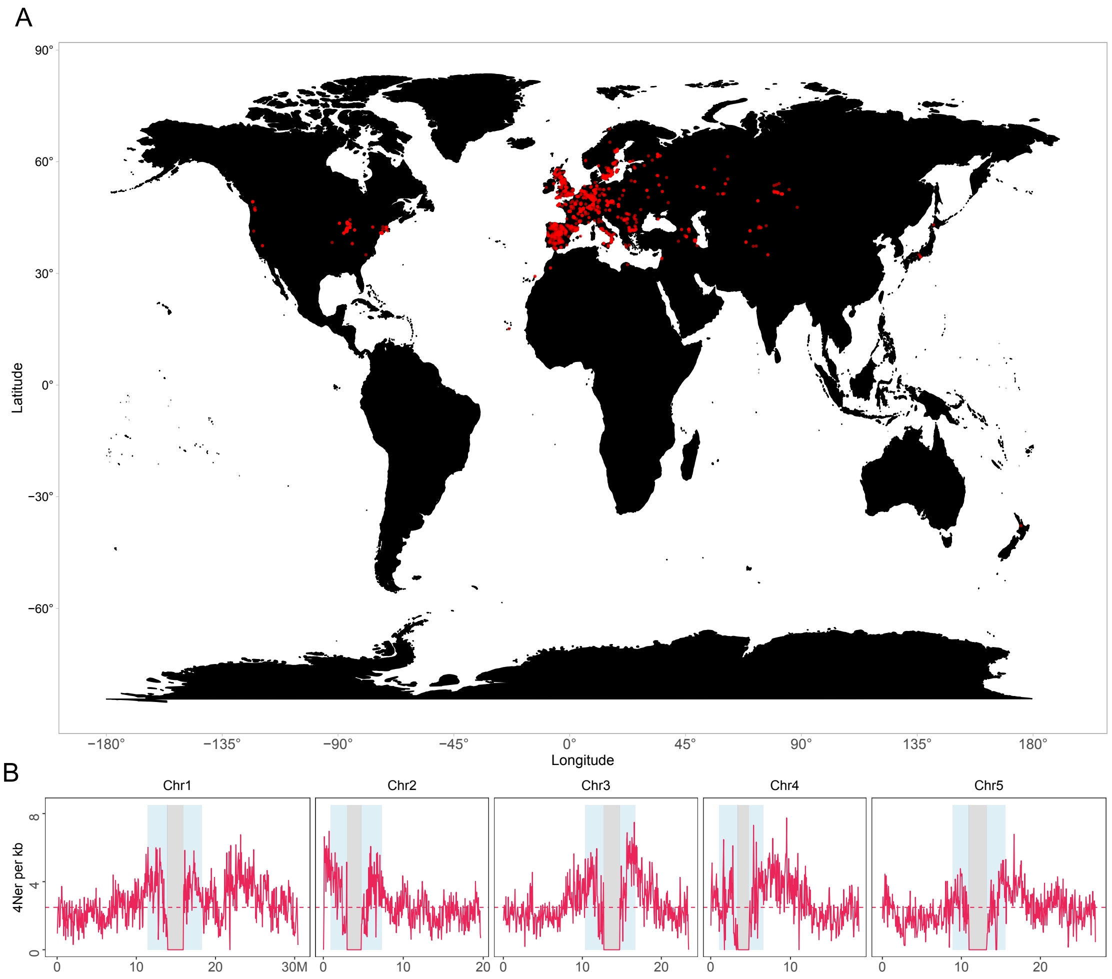


**Supplementary Figure 15. Historical recombination rate in the *Arabidopsis thaliana* genome.** (A) The geographical distribution of the 2,029 Arabidopsis accessions (the 1001 Genomes Project and the RegMap lines, the latitude and longitude for eight accessions were not included). (B) The distribution of historical recombination rates (4Ner per kb) along chromosomes at 50-kb window-scale within 2,029 Arabidopsis accessions. Mean values are indicated by horizontal lines. The pericentromeric and centromeric regions are indicated by grey and blue shading, separately. Source Data are provided as a Source Data file.

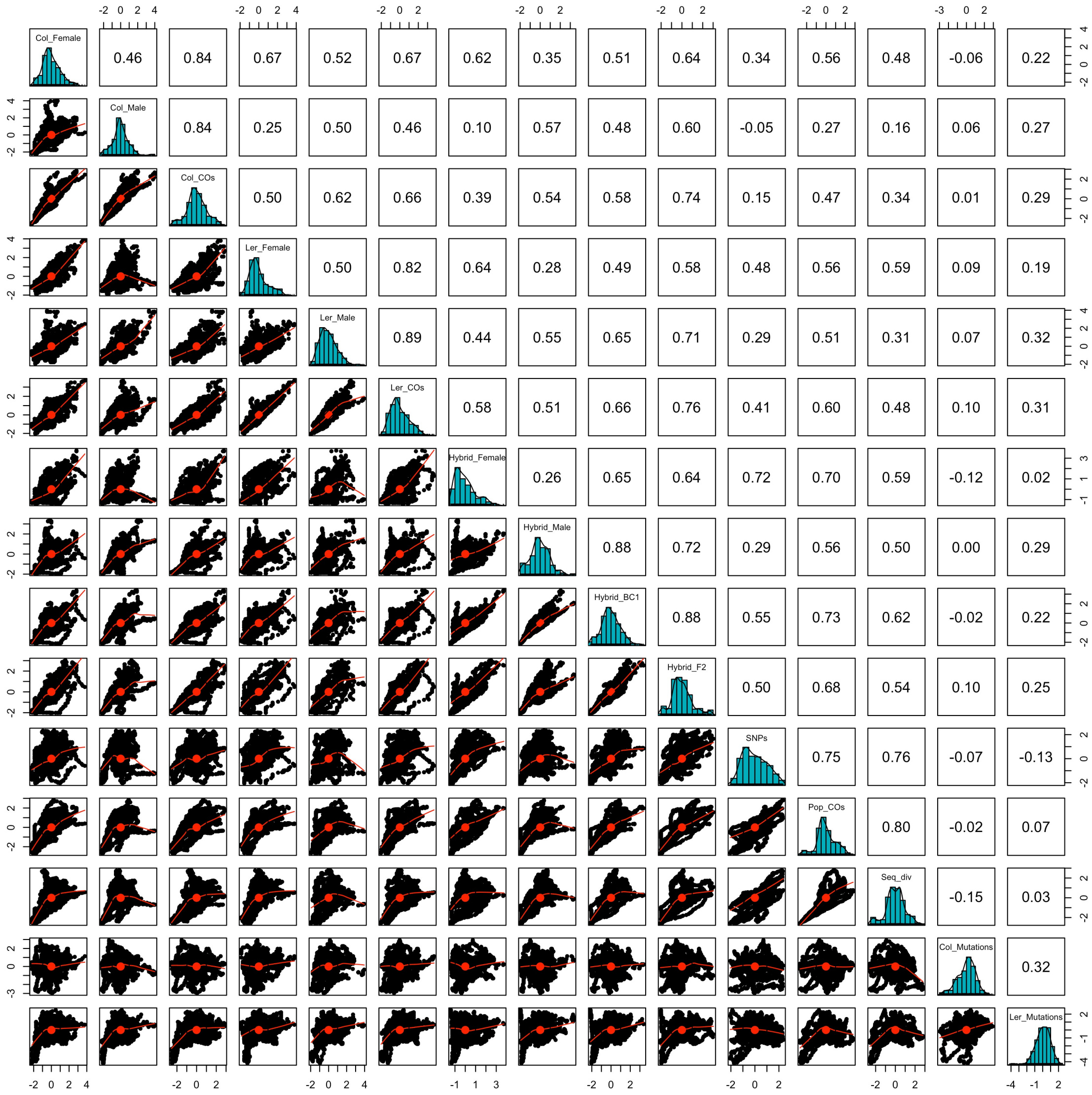


**Supplementary Figure 16. Correlation analysis of CO distribution with polymorphisms.** Spearman's correlation between each pair of features is shown in the upper triangle panel. Source Data are provided as a Source Data file.

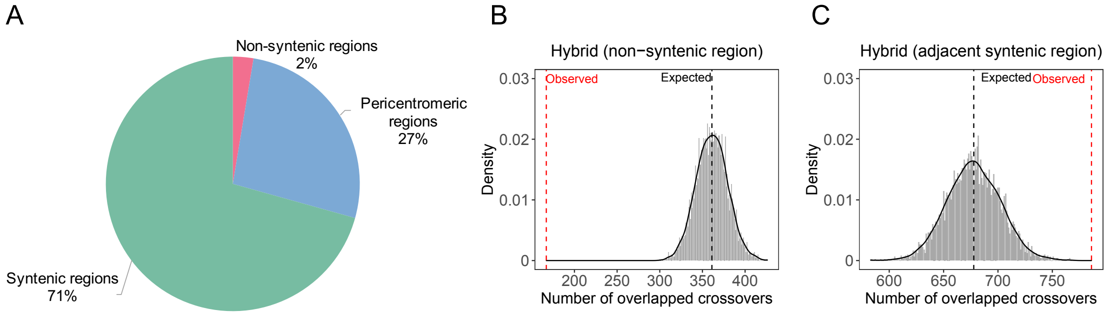


**Supplementary Figure 17. Permutation analysis of the overlap between non-syntenic and adjacent syntenic regions and COs in Col/L*er* F2 hybrids.**

(A) The percentages of non-syntenic and syntenic regions in Col and L*er* and pericentromeric regions. (B–C) Permutation tests were performed to evaluate the overlap between non-syntenic and adjacent syntenic regions and COs in Col/L*er* F2 hybrids, respectively. The adjacent syntenic regions were defined as the up- and downstream regions (same size) of non-syntenic regions. The vertical black dashed lines indicate the expected mean number of overlaps in 5,000 shuffled permutations. The observed number of overlaps is represented by vertical red dashed lines. Source Data are provided as a Source Data file.

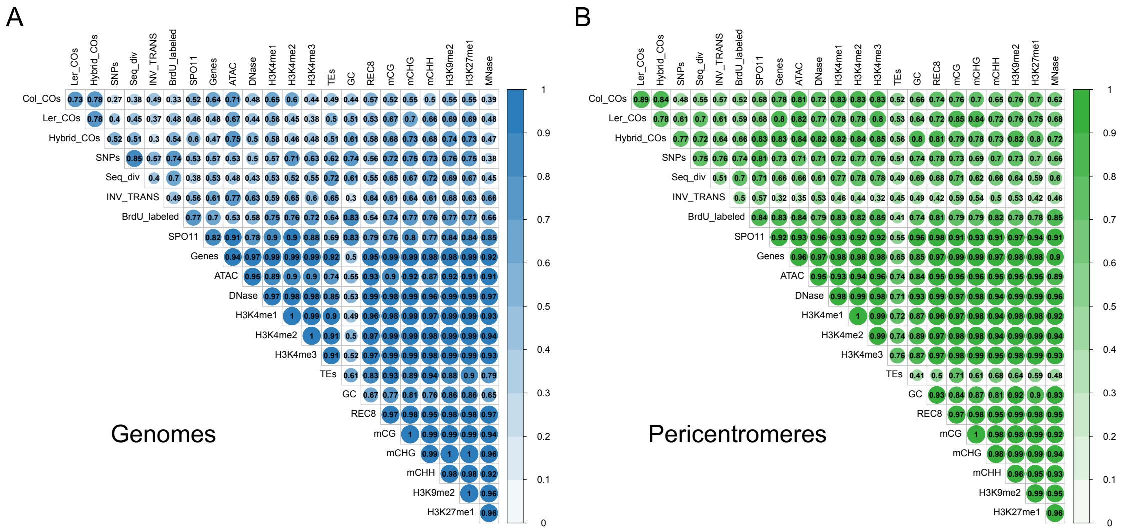


**Supplementary Figure 18. Non-linear correlation coefficient matrices for the distribution of COs, genomic and epigenomic features at genome and pericentromeric scales.**

The non-linear correlation analysis at genome (A) and pericentromeric (B) scale, respectively. Col_COs, Ler_COs and Hybrid_COs (CO landscapes in Col, L*er*, and F2 hybrids), SNPs ( SNPs density between Col and L*er*), Seq_div (sequence diversity in the population of 2,029 Arabidopsis accessions), INV_TRANS (inversions and translocations between Col and L*er*), BrdU_labelled (origins of DNA replication, log2(BrdU/gDNA)), SPO11 (SPO11-1-oligos, log2(oligos/gDNA)), Genes, TEs and GC (gene, TE and GC content density), ATAC and DNase (chromatin accessibility, ATAC-seq and DNase-seq, log2(Tn5/gDNA) and log2(DNase/gDNA)), H3K4me1/2/3, H3K9me2, H3K27me1 (euchromatin, heterochromatin and Polycomb histone marks, ChIP-seq, log2(ChIP/input)), REC8 (cohesin, ChIP-seq, log2(ChIP/input)), mCG, mCHG and mCHH (DNA methylation in CG, CHG and CHH contexts, proportion methylated cytosine), MNase (nucleosome occupancy, MNase-seq, log2(MNase/gDNA)). Source Data are provided as a Source Data file.

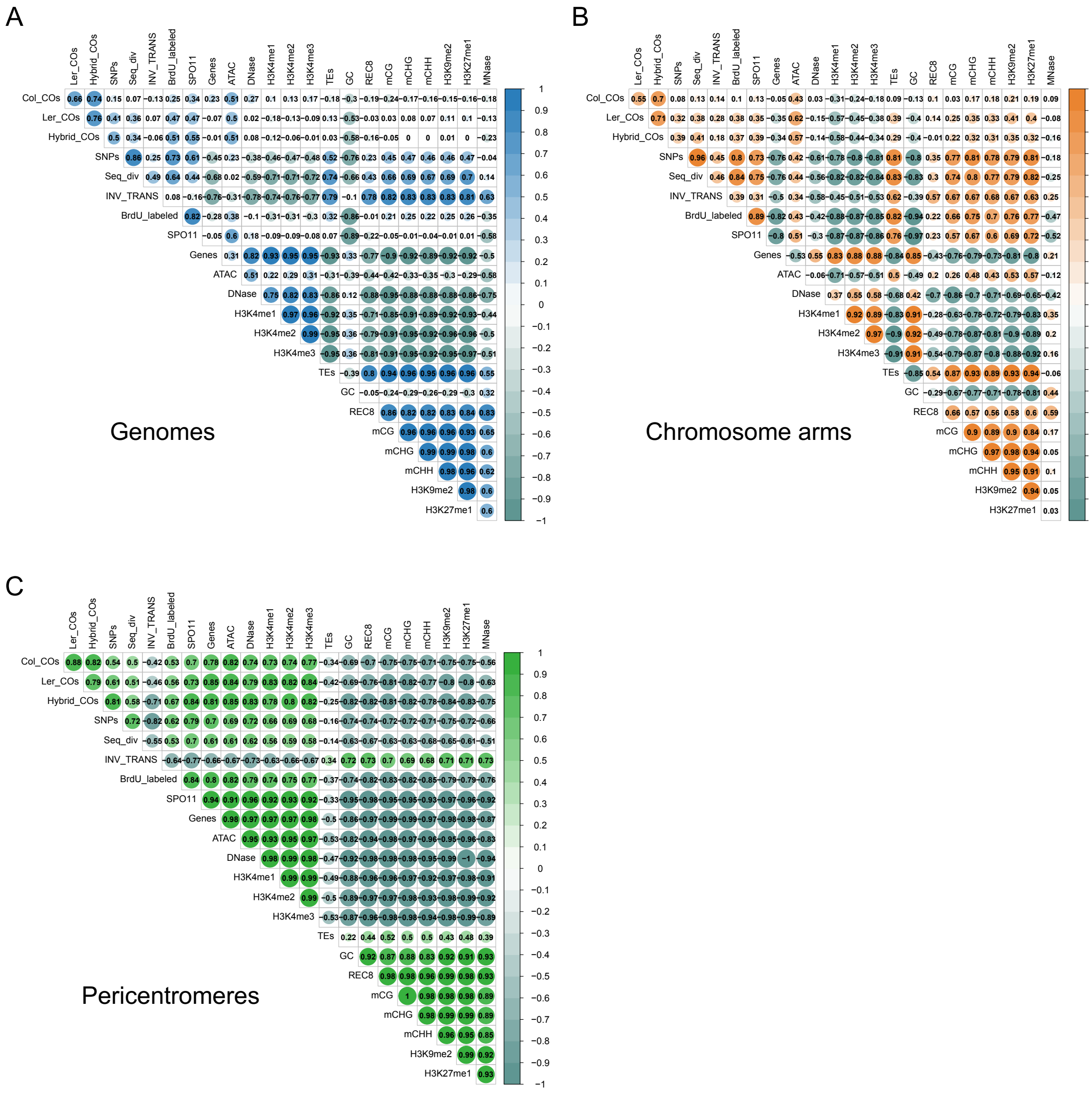


**Supplementary Figure 19. Spearman's correlation analysis for the distribution of COs, genomic and epigenomic features at genome-, chromosome-arm and pericentromeric-scales.** The correlation analysis at genome (A), arm (B) and pericentromeric (C) scale, respectively. Col_COs, Ler_Cos, and Hybrid_COs (CO landscapes in Col, L*er*, and F2 hybrids), SNP ( SNPs density between Col and L*er*), Seq_div (sequence diversity in the population of 2,029 Arabidopsis accessions), INV_TRANS (inversions and translocations between Col and L*er*), BrdU_labelled (origins of DNA replication, log2(BrdU/gDNA)), SPO11 (SPO11-1-oligos, log2(oligos/gDNA)), Genes, TEs, and GC (gene, TE and GC content density), ATAC and DNase (chromatin accessibility, ATAC-seq and DNase-seq, log2(Tn5/gDNA) and log2(DNase/gDNA)), H3K4me1/2/3, H3K9me2, H3K27me1 (euchromatin, heterochromatin and Polycomb histone marks, ChIP-seq, log2(ChIP/input)), REC8 (cohesin, ChIP-seq, log2(ChIP/input)), mCG, mCHG and mCHH (DNA methylation in CG, CHG and CHH contexts, proportion methylated cytosine), MNase (nucleosome occupancy, MNase-seq, log2(MNase/gDNA)). Source Data are provided as a Source Data file.

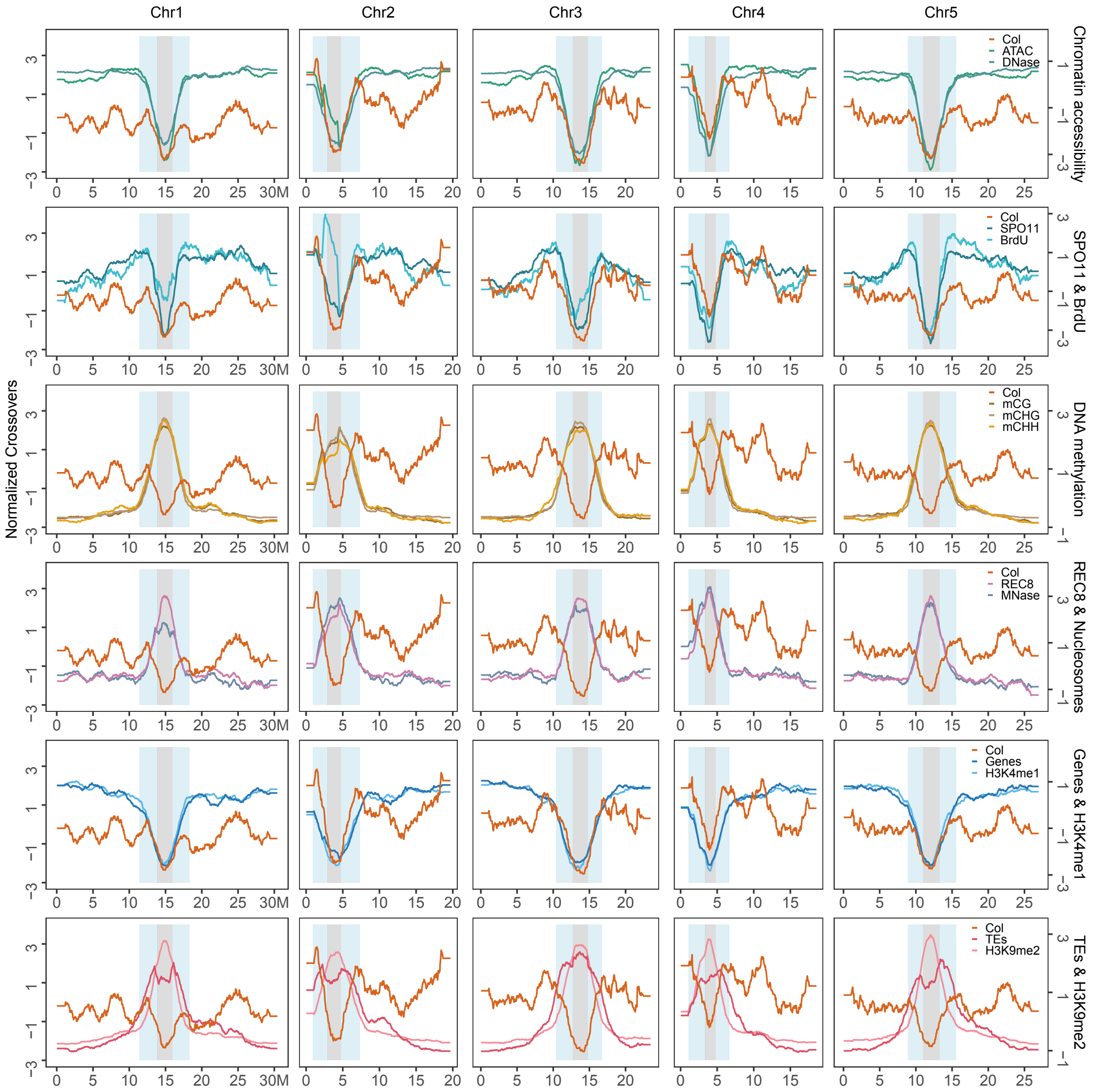


**Supplementary Figure 20. Genomic landscape of Col COs, genomic and epigenomic features.** Sliding window-based distributions (window size 2 Mb, step size 50 kb) were normalized to the same scale. The pericentromeric and centromere regions are indicated by grey and blue shading separately. Col (CO landscapes in Col), BrdU_labelled (origins of DNA replication, log2(BrdU/gDNA)), SPO11 (SPO11-1-oligos, log2(oligos/gDNA)), Genes and TEs (gene and TE density), ATAC and DNase (chromatin accessibility, ATAC-seq and DNase-seq, log2(Tn5/gDNA) and log2(DNase/gDNA)), H3K4me1 and H3K9me2 (euchromatin and heterochromatin histone marks, ChIP-seq, log2(ChIP/input)), REC8 (cohesin, ChIP-seq, log2(ChIP/input)), mCG, mCHG and mCHH (DNA methylation in CG, CHG and CHH contexts, proportion methylated cytosine), MNase (nucleosome occupancy, MNase-seq, log2(MNase/gDNA)). Source Data are provided as a Source Data file.

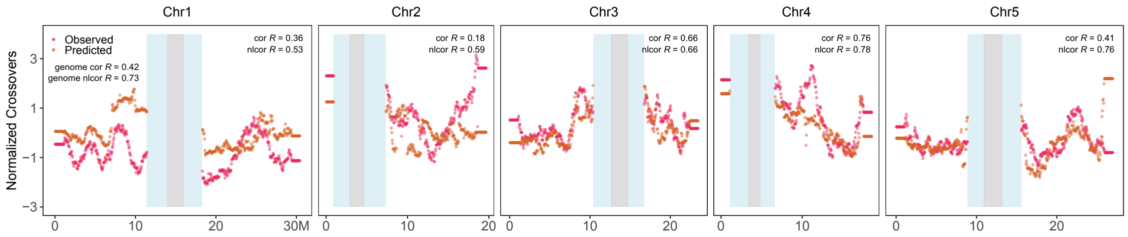


**Supplementary Figure 21. The chromosomal distribution of observed and predicted COs on chromosome arms in Col.**

The CO profiles of individual chromosomes were predicted using profiles of the top six most important features from the other four chromosome arms. The Spearman's and non-linear correlation coefficients between the predicted and observed CO distributions for each and all chromosome arms are indicated, respectively. Source Data are provided as a Source Data file.

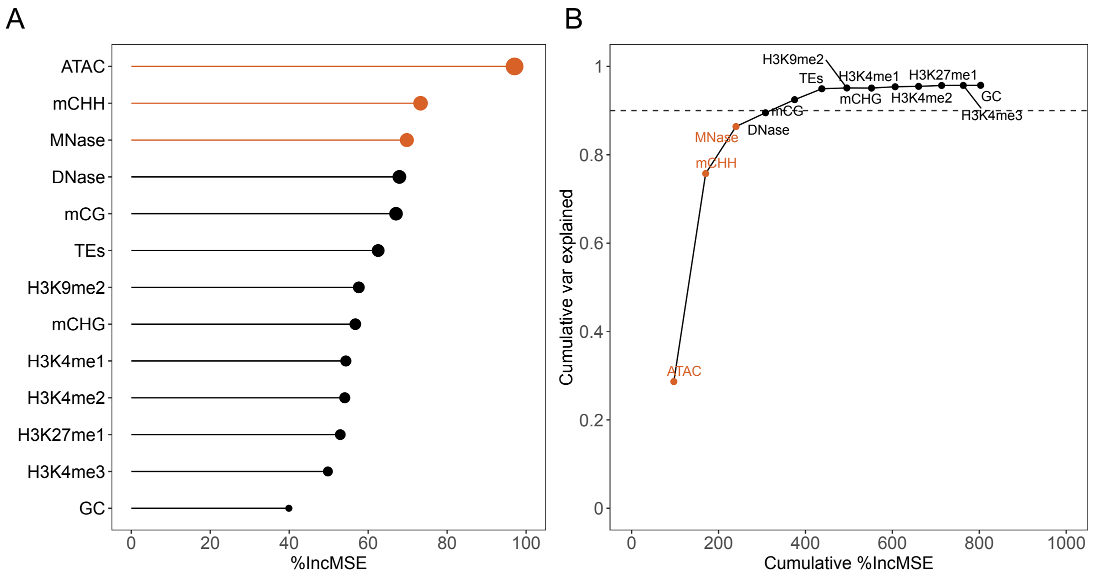


**Supplementary Figure 22. The Evaluation of the importance of genomic and epigenomic features without gene density on chromosome arms in Col.**

(A) The importance of each of the 13 features for explaining the variation in CO distribution at the chromosome-arm scale. The size of points corresponds to the importance. (B) The cumulated proportion of variation that can be explained with the features at the chromosome-arm scale. The top three most important features are coloured. Source Data are provided as a Source Data file.

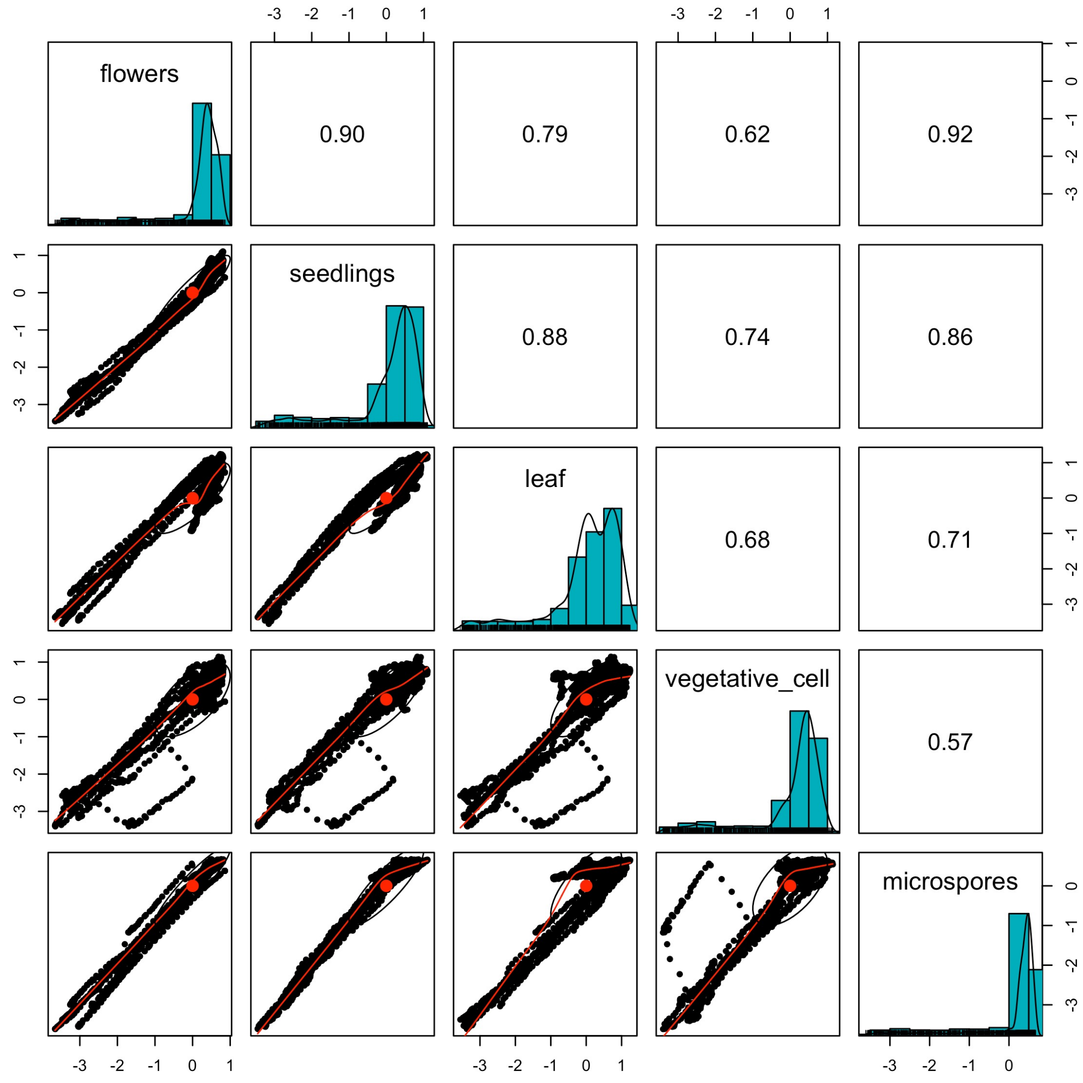


**Supplementary Figure 23. Correlation analysis of the open chromatin profile (ATAC-seq) between tissues in Col.**
Spearman's correlation between each pair of datasets is shown in the upper triangle panel. Source Data are provided as a Source Data file.

## Supplementary Reference


1. Lambing C*, et al.* Interacting Genomic Landscapes of REC8-Cohesin, Chromatin, and Meiotic Recombination in Arabidopsis. *Plant Cell* **32**, 1218-1239 (2020).
2. Choi K*, et al.* Nucleosomes and DNA methylation shape meiotic DSB frequency in Arabidopsis thaliana transposons and gene regulatory regions. *Genome Res* **28**, 532-546 (2018).
3. Zhang WL, Zhang T, Wu YF, Jiang JM. Genome-Wide Identification of Regulatory DNA Elements and Protein-Binding Footprints Using Signatures of Open Chromatin in Arabidopsis. *Plant Cell* **24**, 2719-2731 (2012).
4. Alvarez JM*, et al.* Local Changes in Chromatin Accessibility and Transcriptional Networks Underlying the Nitrate Response in Arabidopsis Roots. *Mol Plant* **12**, 1545-1560 (2019).
5. Zhong Z*, et al.* DNA methylation-linked chromatin accessibility affects genomic architecture in Arabidopsis. *Proc Natl Acad Sci U S A* **118**, e2023347118 (2021).
6. Maher KA*, et al.* Profiling of Accessible Chromatin Regions across Multiple Plant Species and Cell Types Reveals Common Gene Regulatory Principles and New Control Modules. *Plant Cell* **30**, 15-36 (2018).
7. Walker J*, et al.* Sexual-lineage-specific DNA methylation regulates meiosis in Arabidopsis. *Nat Genet* **50**, 130-137 (2018).
8. Costas C*, et al.* Genome-wide mapping of Arabidopsis thaliana origins of DNA replication and their associated epigenetic marks. *Nature Structural & Molecular Biology* **18**, 395-U190 (2011).
9. Bourguet P*, et al.* The histone variant H2A.W and linker histone H1 co-regulate heterochromatin accessibility and DNA methylation. *Nat Commun* **12**, 2683 (2021).
10. Yocca AE, Lu Z, Schmitz RJ, Freeling M, Edger PP. Evolution of Conserved Noncoding Sequences in Arabidopsis thaliana. *Mol Biol Evol* **38**, 2692-2703 (2021).
11. Borg M*, et al.* Epigenetic reprogramming rewires transcription during the alternation of generations in Arabidopsis. *Elife* **10**, e61894 (2021).
