# Supplementary material for: The megabase-scale crossover landscape is largely independent of sequence divergence
Source: Nat Commun. 2022 Jul 2;13:3828. doi: 10.1038/s41467-022-31509-8 (PMC9250513; doi:10.1038/s41467-022-31509-8)
Supplement: Supplementary file 3 — Description of Additional Supplementary Files [file 41467_2022_31509_MOESM3_ESM.pdf]

Description of Additional Supplementary Files

File Name: Supplementary Data 1

Description: The public dataset used in this study

File Name: Supplementary Data 2

Description: COs positions in pure Col

File Name: Supplementary Data 3

Description: COs positions in pure Ler

File Name: Supplementary Data 4

Description: COs positions in Col x Ler backcross population

File Name: Supplementary Data 5

Description: COs positions in Col x Ler F2 population
