# Supplementary material for: The megabase-scale crossover landscape is largely independent of sequence divergence
Source: Nat Commun. 2022 Jul 2;13:3828. doi: 10.1038/s41467-022-31509-8 (PMC9250513; doi:10.1038/s41467-022-31509-8)
Supplement: Supplementary file 9 — Reporting Summary [file 41467_2022_31509_MOESM9_ESM.pdf]

nature portfolio

Corresponding author(s): Raphael Mercier

Last updated by author(s): 2022/06/14

Reporting Summary

Nature Portfolio wishes to improve the reproducibility of the work that we publish. This form provides structure for consistency and transparency in reporting. For further information on Nature Portfolio policies, see our Editorial Policies and the Editorial Policy Checklist.

Statistics

For all statistical analyses, confirm that the following items are present in the figure legend, table legend, main text, or Methods section.

n/a	Confirmed	
☐	☒	The exact sample size (*n*) for each experimental group/condition, given as a discrete number and unit of measurement
☐	☒	A statement on whether measurements were taken from distinct samples or whether the same sample was measured repeatedly
☐	☒	The statistical test(s) used AND whether they are one- or two-sided<br>*Only common tests should be described solely by name; describe more complex techniques in the Methods section.*
☐	☒	A description of all covariates tested
☐	☒	A description of any assumptions or corrections, such as tests of normality and adjustment for multiple comparisons
☐	☒	A full description of the statistical parameters including central tendency (e.g. means) or other basic estimates (e.g. regression coefficient) AND variation (e.g. standard deviation) or associated estimates of uncertainty (e.g. confidence intervals)
☐	☒	For null hypothesis testing, the test statistic (e.g. *F*, *t*, *r*) with confidence intervals, effect sizes, degrees of freedom and *P* value noted<br>*Give P values as exact values whenever suitable.*
☒	☐	For Bayesian analysis, information on the choice of priors and Markov chain Monte Carlo settings
☒	☐	For hierarchical and complex designs, identification of the appropriate level for tests and full reporting of outcomes
☐	☒	Estimates of effect sizes (e.g. Cohen's *d*, Pearson's *r*), indicating how they were calculated

Our web collection on statistics for biologists contains articles on many of the points above.

Software and code

Policy information about availability of computer code

Data collection	No software was used to collect data
Data analysis	All softwares and statistic tests used (described in the Methods) including: BWA (v0.7.15-r1140), inGAP-family (v1.0.0), Tandem Repeats Finder (v4.09), SyRI (v1.2), MADpattern (v1.1), FastQC (v0.11.9), Trimmomatic (v0.38), Cutadapt (v1.9.1), BBMap (35.85), Bowtie2 (v2.2.8), Samtools (v1.9), Sambamba (v0.6.8), bamCoverage (v3.4.3), Bismark (v0.22.0), VCFtools (v0.1.16) and FastEPRR (v2.0), and filter function in stats (v3.6.2) R package, and scale function in base (v3.6.2) R package, and R packages nlcor (https://github.com/ProcessMiner/nlcor), randomForest (v4.6-14), and ggplot2 (v3.3.5) for the most of the visualization.

For manuscripts utilizing custom algorithms or software that are central to the research but not yet described in published literature, software must be made available to editors and reviewers. We strongly encourage code deposition in a community repository (e.g. GitHub). See the Nature Portfolio guidelines for submitting code & software for further information.

Data

Policy information about availability of data

All manuscripts must include a data availability statement. This statement should provide the following information, where applicable:

- Accession codes, unique identifiers, or web links for publicly available datasets
- A description of any restrictions on data availability
- For clinical datasets or third party data, please ensure that the statement adheres to our policy

The raw sequencing data of individuals of Col, Ler inbred lines, and the Col/Ler F1 hybrid can be accessed in ArrayExpress under the accession numbers E-MTAB-11248, E-MTAB-11249, E-MTAB-11250, E-MTAB-11251, E-MTAB-11254, respectively. The public datasets used in this study are provided in the Supplementary Data 1. The list of COs identified in Col, Ler, F1 hybrid (female and male) and F2 hybrid can be found in Supplementary Data 2–5. The Col-0 TAIR10

reference genome is downloaded from the TAIR database https://www.arabidopsis.org/. The sequence polymorphism data of 2,029 Arabidopsis accessions is downloaded from https://figshare.com/projects/Imputation_of_3_million_SNPs_in_the_Arabidopsis_regional_mapping_population/72887. The source data used for generating Figures and Supplementary Figures are provided as a Source Data file.

Field-specific reporting

Please select the one below that is the best fit for your research. If you are not sure, read the appropriate sections before making your selection.

☒ Life sciences ☐ Behavioural & social sciences ☐ Ecological, evolutionary & environmental sciences

For a reference copy of the document with all sections, see nature.com/documents/nr-reporting-summary-flat.pdf

Life sciences study design

All studies must disclose on these points even when the disclosure is negative.

Sample size	Sample size was predetermine aiming at ~300 per population. This is a compromise between cost, and accurate estimation of crossover distribution based on previous work (Capilla et al, PNAS 2021. Blackwell et al, EMBO J 2020. Serra et al, PNAS 2018)
Data exclusions	Two replicates in Col (C and D) were discarded, because the marker analysis showed that one of the F1* was resulting from an accidental selfing and not from a cross. Two additional replicates in Ler were also discarded (C and H), because the number of detected mutation markers (<350) was insufficient for good genome coverage. These two quality controls were pre-established.
Replication	All attempts of replication were successful. The replication are included in the manuscript (A, B, E, F for col;D, E, F, G for Ler)
Randomization	The samples were allocated into groups according to the genotype and sex of the parent (Col, Ler, Col/Ler, male/female)
Blinding	Data collection was done by sequencing on a platform, and thus blindly. Blinding was not feasible during data analysis because sequence analysis used to call crossovers immediately reveals the genetic background (Col, Ler or hybrids).

Reporting for specific materials, systems and methods

We require information from authors about some types of materials, experimental systems and methods used in many studies. Here, indicate whether each material, system or method listed is relevant to your study. If you are not sure if a list item applies to your research, read the appropriate section before selecting a response.

Materials & experimental systems

n/a	Involved in the study	
☒	☐	Antibodies
☒	☐	Eukaryotic cell lines
☒	☐	Palaeontology and archaeology
☐	☒	Animals and other organisms
☒	☐	Human research participants
☒	☐	Clinical data
☒	☐	Dual use research of concern

Methods

n/a	Involved in the study	
☒	☐	ChIP-seq
☒	☐	Flow cytometry
☒	☐	MRI-based neuroimaging

Animals and other organisms

Policy information about studies involving animals; ARRIVE guidelines recommended for reporting animal research

Laboratory animals	The study did no involved animals but laboratory plants.
Wild animals	The study did no involved animals.
Field-collected samples	*For laboratory work with field-collected samples, describe all relevant parameters such as housing, maintenance, temperature, photoperiod and end-of-experiment protocol OR state that the study did not involve samples collected from the field.*
Ethics oversight	*Identify the organization(s) that approved or provided guidance on the study protocol, OR state that no ethical approval or guidance was required and explain why not.*

Note that full information on the approval of the study protocol must also be provided in the manuscript.
